# When “facts” are not facts: what does *p* value really mean, and how does it deceive us?

**DOI:** 10.1007/s10815-020-01751-4

**Published:** 2020-04-07

**Authors:** Caiyun Liao, Andrew L. Speirs, Sierra Goldsmith, Sherman J. Silber

**Affiliations:** 1 Department of Obstetrics and Gynecology, University of Tennessee Health Science Center, Memphis, TN USA; 2 Formerly of Royal Women’s Hospital Melbourne and Melbourne IVF, Melbourne, Australia; 3 Infertility Center of St. Louis, St. Louis, MO USA

**Keywords:** Statistical significance, Multiple comparison, Scientific inference, Frozen embryo transfer, Childhood cancer

## Abstract

The recent paper in JAMA alleging that frozen embryo transfer causes twice the risk of childhood cancer in the offspring is an excellent example of the erroneous use of statistical tests (and the misinterpretation of *p* value) that is common in much of the medical literature, even in very high impact journals. These myths backed by misleading statements of “statistical significance” can cause far-reaching harm to patients and doctors who might not understand the pitfalls of specious statistical testing.

On December 10, 2019, the very prestigious and high impact Journal of American Medical Association (JAMA) published a study titled “Association Between Fertility Treatment and Cancer Risk in Children,” authored by Hargreave et al. [1]. Based on the Danish population-based registry and the Danish Infertility Cohort, this study had the appearance of a statistically sound report, and it stunned the world.

All children born alive in Denmark between 1996 and 2012 were screened for inclusion, with few exclusions due to missing information or neonatal death. 1,085,172 children were included in the study, with an accumulated 12.2 million person-years of follow-up (mean, 11.3 years). In the primary analysis, 90 sets of comparisons were made with diagnosis of infertility, utilization of fertility drugs, and assisted reproductive technology (ART), using fertile women as the reference group. This gave rise to three “statistically significant” hazard ratio (HR) estimates based on a 2-sided *α* of 0.05, pointing to elevated risks of cancers among children born from pregnancies conceived via frozen embryo transfer (FET). The authors wrote, “compared with children born to fertile women, the use of frozen embryo transfer was associated with an elevated risk of childhood cancer (14 cancer cases; HR 2.43 [95% CI (confidence interval), 1.44 to 4.11]”, “mainly due to an increased risk of leukemia (five cancer cases, HR 2.87 [95% CI, 1.19 to 6.93]”.

The media have been in a tumult in response to this study. Internet articles with alarming titles, such as “Freezing embryos doubles risk of IVF kids developing childhood cancer”; “Babies born from IVF using frozen embryos may be more than TWICE as likely to get childhood cancer”; “Children born from frozen eggs twice as likely to develop cancer”; have appeared with frightening alarm. In the methodology and interpretation of this study, there are profound errors which could devastate infertility patients. Many physicians, although skeptical, are not criticizing the paper’s profoundly flawed conclusions, but are merely saying that “the effect is too small to worry about”. This study by Hargreave et al. represents a prime example of the widespread confusion in statistical testing and scientific inference. We wish to address these confusions specifically in regard to this paper, but also for a vast array of similarly misleading papers which do great harm in the name of “statistical significance” which is not actually significant.

## Multiple comparisons: “Let’s Go Hunting”

Hargreave et al. made 90 sets of comparisons in their primary analyses. This is a classic example of “data mining,” also known as “data fishing,” “data dredging,” or “data snooping” [2]. To understand what can arise from such a “shotgun” game with statistics, let us revisit an example we first gave 29 years ago. Twenty-eight consecutive azoospermic men undergoing microscopic epididymal sperm aspiration were studied to determine if having certain letters in their names could “predict” pregnancy [2]. It is not hard to understand that when a wide net is cast to include all the 26 letters for comparison, some dubious findings simply must turn up. Indeed, all the patients who achieved live births had surnames containing the letters G, Y, or N (“GYN positive”), whereas only 29% (6/21) of those who did not achieve live births were “GYN positive”. Fisher’s exact test gave rise to a *p* = 0.003. The idea of using alphabetic letters in the surname to predict pregnancy is so absurd and implausible that all readers will know it cannot be valid despite the glittering *p* value. That is why we did the “study”: to emphasize that *p* values should not be interpreted in isolation. By studying groupings of any three letters in the surnames of these patients (as well as single and two letter groups), we had some 3000 combinations for analysis and were virtually certain to find *p* = 0.003 somewhere. However, in real life, the same abuse of statistics is often far less obvious to the statistically naïve, as in the Hargreave study. It is the uncorrected use of multiple tests for “statistical significance” which is the problem.

A review of the principles of probability and statistical testing will aid the understanding of this issue. According to the American Statistical Association, “a *p*-value is the probability under a specified statistical model that a statistical summary of the data would be equal to or more extreme than its observed value” [3]. Using the Hargreave study as an example, when the incidence rates of childhood cancer were compared, the resulting *p* value refers to the probability that the difference would have been *at least* as large as what they observed, *if* the same study were repeated across many different populations and settings, and *if all* the underlying assumptions were correct. These include the assumptions underpinning the study design, data collection, statistical models, and inference, as well as our assumption that the *p* value was not selected for presentation because it was < 0.05, and that the study design had been reported with completeness and transparency [4]. The *p* value is a parameter that reflects the compatibility between the statistical models and the observed data. A small *p* value could have arisen from inappropriate model specification, flaws in the assumptions during study design, or study bias. In other words, a “significant” *p* value is neither necessary nor sufficient for us to determine that there is a “significant” effect, because the *p* value itself tells nothing about the validity of the study methods. Likewise, with regard to 95% CIs, the 95% refers only to how often 95% CIs computed from very many studies would contain the true value *if all* the assumptions used to compute the intervals were correct [4, 5]. The *p* value and the 95% CI rely heavily on the distribution of the *study* data.

The concept of a 95% CI can be further illustrated with a slightly different approach. If we assume that there is no association between FET and childhood cancers, the null hypothesis will be HR = 1. Imagine us drawing a study sample from the whole population of children and then estimating the HR. We would replace the children back to the population, and repeat the same random sample drawing and HR estimation process 100 times, generating 100 distinct HR estimates, and 100 distinct 95% CIs. If the null hypothesis of “HR = 1” holds, we will be confident that these 95% CIs will cover “1” 95% of the time, and the probability of seeing anything beyond this interval will be less than 5%, *if* all the assumptions underlying the statistical model hold. However, if we do observe a value that is beyond this interval, we will know that either the null hypothesis does not hold, meaning there *is* an association between FET and childhood cancer, *or at least* one underlying assumption is violated, *or* we have run into this finding purely by *chance* (type 1 error).

“Statistics is the science of learning from data, and of measuring, controlling, and communicating uncertainty” [6]. Uncertainty is inevitable as the “truth” is unknown; however, it may be estimated with varying levels of accuracy and precision. “Statistical significance” was originally proposed to indicate the needs for further scrutiny, but with its widespread use, it has often been confused with “scientific importance” [5]. Further, the dichotomous definition of “statistical significance”, often known as “*p* < 0.05”, originated from an arbitrary decision driven by tradition and convenience; it may be used to *screen* for potentially valid assumptions, but should not be equated with scientific conclusions [7]. Therefore, as Greenland commented, the disregard of the underlying scientific principles and “disintegration of *p* value into ‘significant’ and ‘nonsignificant’ is an especially pernicious scientific practice” [4]. Compounding the widespread confusion about “*p* < 0.05” is the question “Is the *p* actually <0.05?” If we estimate the HRs for 90 sets of comparisons, then the probability that *all* our estimates will cover the “true” values under the null hypothesis, at the significance level (*α*) of 0.05 for *each* comparison, will be (0.95)^90^ = 0.0099. Therefore, assuming there is no model misspecification or bias, the probability of us finding *something* “significant” just by chance (type 1 error), and hence rejecting *at least one* null hypothesis, will be 1–0.0099 = 0.99. In other words, in the study by Hargreave et al., they would have found *some* “statistically significant” HR 99% of the time, even if the finding would be as senseless as predicting pregnancy from the letters in the patients’ names.

Several statistical methods have been proposed to reduce the risk of type 1 error in the case of multiple comparisons, assuming the study is otherwise free of bias. Among these methods is the Bonferroni correction, which is a more conservative approach that minimizes spurious findings by chance. Using the Hargreave study as an example, if we were to set the significance level such that we may reject a null hypothesis by chance 5% of the time with *all* the planned comparisons lumped together, then for *each* individual comparison, the *α* should have been 0.05/90 = 0.00056, instead of 0.05. With such corrections, what was reported in the Hargreave study would not have been statistically significant based on the *α* defined a priori, unless they could show a *p value* less than 0.00056.

Similarly, in clinical trials, various methods have been developed to restrict the *α* for each interim analysis (*α* spending), in order to control the overall type 1 error rate [8]. Without these adjustments, “statistically significant” results will always arise by chance if enough comparisons are made, and a trial may very well be incorrectly stopped prematurely based on simply random findings. Therefore, the fallacy of multiple comparisons cannot be addressed by simply changing the design to a prospective one, be it an observational study or a clinical trial. It is just as easy to make multiple comparisons prospectively, and only report findings that are “statistically significant”.

It is thus obvious that even if there is no other bias, the combination of a “let’s go hunting” approach and the lack of proper statistical correction in the Hargreave study have given rise to the dubious *p* < 0.05, deceiving not only the media and the lay public, but also many physicians. In their Statistical Methods section, the authors state “Because no adjustment was made for multiple comparisons, the findings should be considered exploratory given the large number of treatment subgroups that are being compared.” But if readers did not examine the supplementary tables carefully, it would have gone unrecognized that the vague statement of “the large number of subgroups” actually meant 90 tests for statistical significance. With so many comparisons, it was inevitable that some “statistically significant” but probably meaningless effect would be found. Responsible publishing dictates candid disclosure of the number of comparisons attempted, transparent report of the actual statistical adjustment made to address multiple comparison, honest recognition of the uncertainty in statistical findings, modest reporting of study results and their “practical” significance, and open acknowledgement of all other study limitations.

## Association versus causation

In 1950, Richard Doll and Austin Bradford Hill published the famous study on the causal relationship between smoking and lung cancer, which has been the prime example of causal inference in epidemiological studies [9]. However, despite this breakthrough 70 years ago, misuse of statistics and confusion between *association* and *causation* have persisted to date. In 1965, Bradford Hill addressed these issues in his seminal paper, “*The Environment and Disease: Association or Causation?”* [10]. In this paper, Bradford Hill proposed nine features of an association that are consistent with a causal relationship, which are relevant in the interpretation of the Hargreave study and many others.

1. Strength of the association between the exposure and the outcome, as measured by statistical parameters, such as relative risk (RR), odds ratio (OR), incidence rate ratio (IRR), HR, etc. Bradford Hill considers this to be the most important feature of a causal association. For example, the causal association between female age and infertility can be demonstrated with the drastic decrease in the live baby rate per oocyte of 26% in patients younger than 35, to 1% of those who are older than 42 [11].
2. Consistency across studies *properly* performed in different scenarios and at different times. For example, the association between age and infertility was also supported by national ART surveillance data in the U.S. and Australia/New Zealand [12, 13], among many other regions. Note that bias in the original study cannot be addressed by simply repeating it, without correcting fallacies and errors in its design and interpretation.

Recognizing the importance of the consistency criterion, Hargreave et al. quoted four previous studies examining the association between FET and childhood cancer in the Discussion section. Three of these studies did not support such association. They then wrote: “one study reported an elevated HR for any type of cancer (HR, 1.80 [95% CI, 0.65-4.95]) and another study reported an elevated HR for hepatoblastoma (HR, 5.24 [95% CI, 0.13-29.21]).” Note that the 95% CIs for both HRs crossed 1, and therefore *neither* of these HRs was statistically significant. It is a blatant error to say that this represents “an elevated HR”. In other words, while Hargreave et al. misleadingly claimed that at least one out of four previous studies supported their FET-childhood cancer theory, in fact *none* of them provided such support. We find it surprising that such a bold misinterpretation went unnoticed during the review and publication process for JAMA.

The importance of the consistency feature was recently reiterated by Hubbard, Haig, and Parsa: “*Scientific* inference is a far broader concept than *statistical* inference… A major focus of scientific inference can be viewed as the pursuit of *significant sameness*, meaning replicable and empirically generalizable results among phenomena. Regrettably, the obsession with users of statistical inference to report *significant differences* in data sets actively thwarts cumulative knowledge development.” [14].

(3) Specificity of the association, such as *M. tuberculosis* causing tuberculosis in humans, and human chorionic gonadotropin or luteinizing hormone (LH) triggering ovulation.
(4) Temporality, meaning the cause should precede the outcome in time. For example, in a spontaneous ovarian cycle, the LH surge precedes ovulation.
(5) Dose-response relationship. This is corroborated by the continuous decline in women’s fecundity as they age [11–13].
(6) Biological plausibility, as is concept of chromosome 21 nondisjunction leading to Down syndrome, has been plausible for the medical community.

While we acknowledge that this may be limited by the biological knowledge of the day and can evolve over time, so far, there is no good evidence supporting an association between FET and childhood cancer. In the Introduction section of the Hargreave study, there was only one study that seemed to support the biological plausibility of this hypothesis: “It has been suggested that the use of fertility treatment increases the risk of cancer in children, possibly through epigenetic changes brought on by the use of fertility drugs, ART, or both.” However, this turned out to be a paper Hargreave et al. published in *Fertility and Sterility* in 2013 [15]. The lack of evidence becomes evident when the one and only study the authors could cite came from themselves.

The effort of Hargreave et al. to rationalize their claims did not stop there. In the Discussion section, they went on to state that estrogen was “an established carcinogen”, and progesterone was “reasonably anticipated to be a human carcinogen”. Such statements are dangerously misleading, and in fact are also contradictory to their findings that fertility drugs were not associated with childhood cancer.

(7) Coherence with generally known facts. For example, the chromosome 21 nondisjunction phenomenon observed in genetic studies is coherent with the higher incidence of Down syndrome among children born to older women. Likewise, in the Hargreave study, the coherence criterion may be met if epidemiological data do show an increased risk of childhood cancer associated with FET, and bench studies also reveal certain pathophysiological pathways leading from FET to oncogenesis.
(8) Supporting evidence in clinical trials or experiments. For example, the causal association between the transfer of multiple embryos and multiple pregnancies is supported by randomized controlled trial data, where transferring two embryos in younger women led to drastically more multiple births (33.1% versus 0.8%), without increasing the overall live birth rates with a comparable magnitude [16].
(9) Analogy found in other relevant areas. Understanding the association between maternal age and trisomy 21, we would be ready to accept similar associations between age and other forms of aneuploidy.

As one may see from the list above, statistical significance is necessary, yet insufficient, to define causation. The comments by Bradford Hill in 1965 on this matter were as relevant then as they are today [10]:

“*No formal tests of significance can answer those questions. Such tests can, and should, remind us of the effects that the play of chance can create, and they will instruct us in the likely magnitude of those effects. Beyond that they contribute nothing to the ‘proof’ of our hypothesis*.”

A well-contemplated conceptual framework and an a priori hypothesis are central to the validity of any epidemiological study. The conceptual framework is represented by all the epidemiological processes that can give rise to an observed association, and all the biological processes that can make such associations reasonable. In constructing the conceptual framework, one must consider confounding, selection bias, mediation, effect modification, generalizability, and the contrast between clinical versus statistical significance.

## Confounding

Confounding is one of the fundamental issues in observational studies, and it is essentially an issue of non-exchangeability. In order to prove an association between FET and childhood cancer, ideally, we would hope to have two groups of children who are identical (or “exchangeable”) in every feature, except for the utilization of FET in the index pregnancies. However, as common sense tells us, this is impossible in human studies. The next to ideal method to address confounding is randomization, which is often unfeasible due to logistical or ethical reasons. Statistical models that adjust for confounders may mitigate some of the bias, *if* factors that contribute to the non-exchangeability have been adequately accounted for. In the Hargreave study, the only covariate included in the main models was the year of birth categorized in 5-year intervals. To suggest that this represents an adequate control of confounding is to assert that the comparison groups were indifferent in all the other aspects. That is manifestly implausible.

Since the comparisons were made between women who conceived through FET versus those who conceived spontaneously, the two groups differed not only in the usage of FET, but also distributions of parental age, diagnosis of infertility, use of fertility drugs and ART, embryo cryopreservation, thawing, and transfer. Even within women who were diagnosed with infertility, the etiologies could have been male factor, pelvic factor, anovulation, endometriosis, uterine anomalies, just to name a few. The severity of infertility also differed to some extent among women who eventually conceived spontaneously, versus those who required ART to conceive. Couples undergoing ART are, on average, older than those who have no fertility problems. This is demonstrated in the Hargreave study where 50.7% of the children of the fertile couples were born to women aged under 30, and 14.8% were born to the oldest of the three age groups (> 34). This is very different from the ART group, where only 27.4% of the children were born to women younger than 30. The distribution of paternal ages showed a similar pattern. Since advanced parental age is associated with pediatric cancers [17, 18], it is an obvious confounder that has not been accounted for in the Hargreave study. Moreover, as 35 and 40 are commonly used cutoffs with important implications in reproductive biology, the age stratification would have been much more appropriate had it included two additional age categories, namely 35–39, and ≥ 40. In addition, parental age should ideally be measured at the time of embryo creation, rather than the time when the children were born, as in the Hargreave study. With FET, this could be several years different.

Along the same line, the children’s age should have been studied as a confounder with more granularity. First, it has been shown that the proportional distributions of pediatric cancer types vary by age [19]. For example, leukemia, which was a major cancer type studied by Hargreave et al., was the dominant cancer diagnosis among children under 10, and became less common in the older age groups [19]. Secondly, due to the improvement in efficiency and popularity of FET over time, there would have been a higher proportion of children who were born from FET in the later calendar years. In other words, the FET group would have had more younger children, who were at higher risk of developing cancers such as leukemia than the control group. This was supported by the data presented by Hargreave et al.: the average length of follow-up since birth was 30,260 person-years/3356 children = 9.02 years in the FET group, and 10,385,749 person-years/910,291 children = 11.4 years, among children born to fertile women. Regardless, children’s age was not included in the main or sensitivity analyses of Hargreave et al.

In addition, donor (egg, sperm, or embryo) ART cycles should have been accounted for, as the genetic predisposition of children born from these cycles is strongly related to that of the donors. The age of the donor should be used in the regression analysis, but was not used in the Hargreave study. Was this appropriate? According to the Centers for Disease Control and Prevention, 9% of the ART cycles in the United States (U.S.) in 2016 intended to use eggs from a donor [12]. In 2014, donation and recipient cycles accounted for 5.1% of all treatment cycles in Australia and New Zealand [20]. We would therefore assume that egg, sperm, or embryo donation also contributed to a significant proportion of the ART cycles in Denmark, and therefore should have been accounted for in the regression analyses. If this were impossible, the donor cycles should have been excluded altogether, or the authors should have explained why they remained.

The indications and techniques for FET from 1996 to 2012 have evolved remarkably [21]. During the era of the Hargreave study, FETs were largely performed after failed fresh embryo transfers. Due to embryo selection practice, the genetic features of the frozen-thawed embryos in that era could be different from those of the embryos selected for fresh transfers, and embryos that were all frozen for planned embryo banking (“freeze all”). Further, “freeze all” could have been indicated for cancer fertility preservation, prevention of ovarian hyperstimulation syndrome (OHSS), etc. It is possible that any of these indications may confer discrete susceptibility to cancer in the offspring, and hence lead to non-exchangeability and confounding. Generalizability of the study findings to today’s practice is limited by changes in the landscape, such as the increasing popularity of “freeze all” cycles, and the wide adoption of vitrification.

**Fig. 1:**
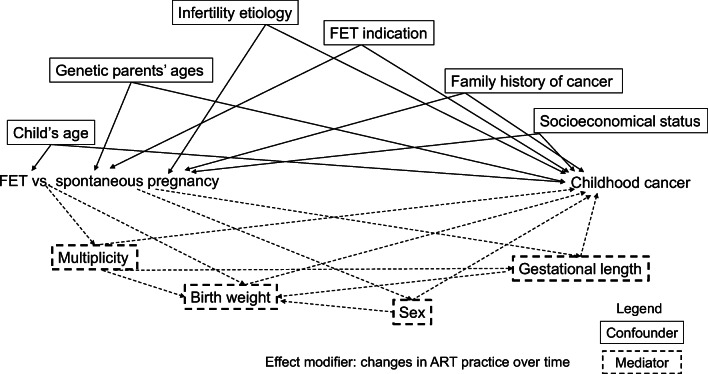
A sample directed acyclic graph (DAG) demonstrating a proposed causal network spanning from frozen embryo transfer (FET) to childhood cancers. In general, in a DAG, confounders are common causes of both the exposure and the outcome. Mediators are effects of the exposure and causes of the outcome. The arrows illustrate the directions of the causal relationships. More complex concepts, such as collider stratification bias, which underlies selection bias, can be readily illustrated on a DAG. A “collider” is a common effect of both the exposure and the outcome. For example, birth weight is a “collider” between FET and sex. Therefore, if one studies the association between FET and sex, and conditions on birth weight, a spurious connection may emerge between FET and sex. An effect modifier is the change in ART (assisted reproductive technologies) practice, which can lead to different associations between FET and childhood cancer over time

It is obvious at this point that spanning from FET to childhood cancer is a conceptual framework that is far too complex for the simple appraisal Hargreave et al. presented. Systematic evaluation of confounding, mediation, selection bias, and even effect modification may be facilitated with directed acyclic graphs (DAG) [22]. A sample DAG is provided to illustrate some of the confounders and mediators that could have complicated the causal pathway between FET and childhood cancer (Fig. 1).

Despite these utter biases that are impossible to rationalize, Hargreave et al. attempted to justify their analyses with purely statistical claims. “Only year of birth… was included in further analyses because none of the other factors changed the risk estimate by more than 10%.” The magic “10%” cutoff is completely empirical, and may play a role when there is no other way to determine if certain factors are indeed confounders. However, researchers must first identify confounders by carefully reviewing the subject knowledge. Confounders thus found must be accounted for in statistical modeling, even if they do not seem to be *statistically* correlated with the exposure or the outcome. Consider the association between diabetes and endometrial cancer as an example. A systematic review of the subject knowledge will reveal age, parity, obesity, smoking, polycystic ovary syndrome, etc., as confounders [23], which must be addressed regardless of the statistical features of the study population.

Hargreave et al. did perform a post hoc calculation of the *E*-value to prove that *statistically* the effect of residual confounding was negligible. Introduced in 2017 [24], *E*-value is a relatively new concept even for clinical epidemiologists, let alone the statistically naïve. Expressed on the risk ratio scale, *E*-value represents the minimal strength of association the unmeasured confounder needs to have with the exposure and the outcome, in order to explain away the observed association. If the *E*-value is high, it means it will be difficult to explain away the observed association, which is therefore robust. But the validity of the *E*-value is conditional on adjustments for the known confounders, which was not done. It is therefore not a panacea for the gross confounding bias in the Hargreave study.

To support their primary findings, Hargreave et al. also performed a sensitivity analysis by adding sex, birth weight, gestational length, and multiplicity as the “intermediate factors”, and changed the reference group to women who conceived with “fertility assistance”. This led to another 99 sets of comparisons and similar findings as those in the primary analyses. “Intermediate factors”, also known as “mediators”, are factors that may have mediated the causal pathway leading from the exposure to the outcome (Fig. 1). For example, the number and quality of oocytes can mediate the causal pathway leading from age to live birth. It is *not* synonymous with confounding, which, as we discussed above, is the dissimilarity among comparison groups. Therefore, by performing sensitivity analyses as such, Hargreave et al. did not address the critical issue of confounding in their primary analyses. Sensitivity analyses that repeat the same errors will not validate the fundamentally spurious findings in the primary analyses.

As the saying goes, “We don’t want to compare apples to oranges.” In observational studies, properly designed regression models may address *most* of the confounding, such that even though we have not randomized the study participants (and therefore non-exchangeability will persist to certain extent), we will at least be comparing grapefruits to oranges. Otherwise, we could end up comparing dinosaurs to oranges, in which case, the conclusion will not be valid no matter how impressive the *p* value may be. As Bradford Hill critiqued, test of significance should only be a guide, rather than a rule, and one should never let the glitter of the *p* “divert attention from the inadequacies of the fare” [10].

## Statistical versus clinical significance

As discussed above, the *p* value is largely driven by the unknown true value of the population, which we attempt to estimate, as well as the distribution of study data. A large sample size increases the precision of the estimate and the likelihood of this estimate being *statistically* significant. This is a common phenomenon in large registry studies, as in the study by Hargreave et al., where *14* cancer cases among 3356 children born from FET pregnancies actually gave rise to *statistically* significant estimates. But such a small number of cases could not have given rise to a *p <* 0.05, had the authors not taken a “shotgun” approach of multiple comparisons and ignored the confounders. Further, when the baseline childhood cancer risk was 17.5 per 100,000 person-years, a “two-fold” increase in such risk actually translated into 26.9 additional cancer cases per 100,000 person-years. The number needed to harm, which is the inverse of the absolute risk difference, was 3717.5 (100,000/26.9) person-years. *Even if* the analysis in the original study was valid, hundreds or thousands of FETs would have to be avoided in order to “prevent” one case of childhood cancer. The small magnitude of this dubious “risk” is in contrast to the numerous benefits of FET, such as unprecedented flexibility in ART treatment, drastic reduction in the risk of OHSS and multiple pregnancies, the opportunity for embryo banking, and preimplantation genetic testing [21, 25]. The benefit and risk ratio in this case appears to be self-evident, and the *dubious statistical* significance has not translated into any *probable clinical* significance. But aside from the obvious lack of “clinical significance,” in fact the Hargreave study also lacks the “statistical significance” that it claims.

## The devastating ramifications

With data misinterpretation, a speciously sound-appearing methodology may produce misleading conclusions with devastating results to society [26]. While the misinterpretation might have been an act of innocence, its ramifications can be profound and harmful. In 1965, Bradford Hill had warned us, “in passing from association to causation I believe in ‘real life’ we shall have to consider what flows from that decision” [10]. While Hargreave et al. did not exactly equate association with causation, they seem to have at least implied it to most readers by phrasing such as “compared with children born to fertile women, the cancer risk increased among children born after the use of frozen embryo transfer”, and “children born after the use of frozen embryo transfer had a statistically significantly higher risk of leukemia, …and sympathetic nervous system tumors… than children born to fertile women.” To understand the scale of damage these unfounded claims can cause, we may use the U.S. data as an example. In 2016, there were 263,577 ART cycles performed in the U.S., 32.7% of which used frozen embryos from nondonor eggs, 5.1% used frozen donor embryos, and *25%* of the cycles were started with the intent to freeze *all* resulting eggs or embryos for potential future use [12]. A spurious assertion that connects FET to childhood cancer will likely cause undue guilt, anxiety, and stress among parents of children who were conceived via FET and diagnosed with cancer later in life, as well as millions of patients worldwide who plan to undergo, or have undergone, embryo cryopreservation and transfer. Spurious associations as such may lead to unjustified rejection of FET, a technology that has transformed infertility treatments and become essential to today’s practice.

The fallacies, errors, and confusions Austin Bradford Hill started to tackle 70 years ago remain just as outrageous today. As two of the coauthors stated 29 years ago, “It is *not* that statistical methods can be used to prove almost anything, but rather that gross abuses may deeply mislead the non-statistician.” [2]. In his 1994 commentary *The Scandal of Poor Medical Research*, Douglas Altman observed, “Huge sums of money are spent annually on research that is seriously flawed through the use of inappropriate designs, unrepresentative samples, small samples, incorrect methods of analysis, and faulty interpretation… Responsible medical journals invest considerable effort in getting papers refereed by statisticians… Unfortunately, many journals use little or no statistical refereeing - bad papers are easy to publish.” [27].

Basic statistical literacy is essential in preventing myths, just as vaccines are essential in preventing the spread of infectious diseases. We all play a role in rising to the challenge. As Altman said, journal editors and reviewers should contribute their due diligence as the “fire fighters” against flawed research studies, balancing potentially important findings with the knowledge of study limitations, statistical uncertainty, need for confirmation studies, and the media’s tendency to omit subtle technical caveats. Clinicians, the second gatekeepers of research information, “need not be experts in statistics, but they should understand the principles of sound methods of research.” [27]. To meet this goal, much remains to be done to integrate *scientific* inference with medical school, residency, and fellowship curriculums.

It is the responsibility of leading medical journals, such as JAMA, to evaluate and reject statistically unfounded studies, even though the specious conclusions they generate may draw headlines and elevate the “impact factor.” When even high impact clinical journals fail us, we must be vigilant in not accepting unsound conclusions.
